# Management outcomes of prolactinoma: a retrospective study from Southern Iraq

**DOI:** 10.25122/jml-2025-0050

**Published:** 2025-09

**Authors:** Hasan Falah Alobaidy, Haider Ayad Alidrisi, Khulood Abed Reman, Qusay Alzajaji, Ibrahim Hani Hussein, Abbas Ali Mansour

**Affiliations:** 1 Al-Hassan Metabolism, Endocrine, and Diabetes Center, Karbala, Iraq; 2 Department of Medicine, Faiha Specialized Diabetes, Endocrine, and Metabolism Center, College of Medicine, University of Basrah, Basrah, Iraq

**Keywords:** pituitary, prolactinoma, hyperprolactinemia, dopamine agonist, transsphenoidal pituitary surgery

## Abstract

Prolactinoma is the most common pituitary adenoma. This study aims to assess the clinical presentation, treatment modalities, and outcomes of patients with prolactinomas and to identify factors that predict remission. We conducted a retrospective single-center study including patients with prolactinoma. Data from medical records were analyzed to correlate patient demographics, clinical presentation, serum prolactin (PRL) levels, and adenoma size on MRI, both at diagnosis and after initiation of dopamine agonist (DA) therapy, with treatment outcomes. A total of 205 patients were included in the study. The mean age of the cohort was 34.8 ± 12.4 years, with a female-to-male ratio of 1.5:1. Oligomenorrhea/amenorrhea was the most common presenting symptom, occurring in 112 of 122 women (91.8%). Macroadenomas accounted for 117 of 176 adenomas (66.4%). Initial treatment consisted of DA therapy in 149 patients, transsphenoidal pituitary surgery in 41 patients, and gamma knife radiosurgery in five patients. A total of 148 patients continued DA therapy at our center. After one year of DA treatment, significant adenoma shrinkage (>30%) was observed in 23 patients (34.3%), while complete adenoma disappearance occurred in six patients (8.9%). At 24 months, 25 of 88 patients (28.4%) achieved remission. Baseline PRL <10,638.2 mIU/L (500 ng/mL) and the presence of microadenoma independently predicted remission. DA therapy remains the cornerstone of prolactinoma treatment in our region and is very effective in normalizing PRL levels, shrinking adenomas, and improving clinical symptoms; DA can be used even in cases of giant prolactinomas.

## Introduction

Hyperprolactinemia is a common disease in clinical practice and has several physiological, pharmacological, and pathological causes. Among the pathological causes, lactotroph adenoma (prolactinoma) is the most common etiology [1]. Microprolactinomas, defined as tumors smaller than 1 cm, occur more often in women, particularly during the third to fifth decades of life [2]. Clinically, prolactinomas typically present with oligomenorrhea or amenorrhea and galactorrhea in women, while erectile dysfunction is the predominant symptom in men. Infertility and decreased libido are common in both sexes [3]. Clinically nonfunctioning pituitary adenomas with suprasellar expansion are a common cause of hyperprolactinemia [4]. Hyperprolactinemia may be found in 8% to 43% of patients with hypothyroidism [5].

Prolactinoma most commonly affects women aged 20 to 50 years, with a gender ratio 10:1 [6]. In this demographic, microadenomas are more prevalent, whereas men are more likely to present with larger adenomas. The discrepancy could be explained by the clinical presentation, as female patients tend to seek medical assessment earlier [7]. Dopamine agonists (DA) are the cornerstone treatment for prolactinomas, resulting in the normalization of prolactin (PRL) levels, adenoma shrinkage, and restoration of gonadal function [2]. The common side effects of DA are dyspepsia, vomiting, and mild dizziness [7]. Mood changes and impulse control disorders may occur with DA therapy in patients with no history of psychiatric disorder [8]. Transsphenoidal pituitary surgery (TSS) can normalize PRL levels in up to 93% of patients with microadenomas and 75% of those with macroadenomas [9]. Invasive or large prolactinomas are often treated with DA, while TSS is reserved for spontaneous or dopamine agonist-induced CSF rhinorrhea [10]. Prolactinoma in men is often large and invasive, occasionally reaching giant size. They commonly present with hypogonadism and can cause mass effects such as hypopituitarism and vision impairment [6].

This study aimed to assess the clinical presentation, treatment modalities, and outcomes of patients with prolactinomas and to identify factors that predict remission.

## Material and Methods

This study employed a retrospective analysis of data from the electronic database of the Faiha Specialized Diabetes, Endocrine, and Metabolism Center (FDEMC) covering the period from January 2012 to April 2024. Relevant data were extracted into Microsoft Excel and subsequently analyzed at FDEMC between June and August 2024.

We included all patients aged 16 years or older with a confirmed diagnosis of prolactinoma. Prolactinoma was diagnosed based on the presence of elevated serum PRL and an identifiable pituitary adenoma on magnetic resonance imaging (MRI) in the context of clinical symptoms. This study excluded patients with prolactinoma whose records were missing key data or MRI results; those with hypothyroidism, liver disease, or renal insufficiency; and patients on a medication regimen that raised PRL levels.

**Figure 1:**
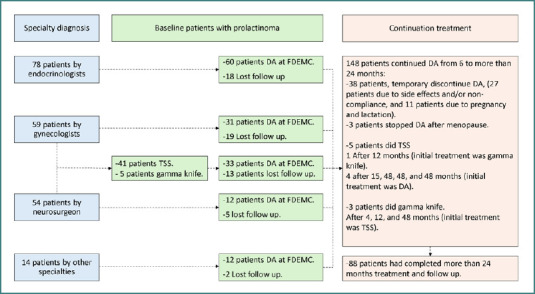
Flowchart showing the study method DA, Sopamine agonists; TSS, transsphenoidal pituitary surgery; FDEMC, Faiha Specialized Diabetes, Endocrine, and Metabolism Center.

A total of 205 patients with prolactinoma were identified at the center. Of these, 78 were diagnosed directly by endocrinologists at the FDEMC, while the remaining patients were referred by gynecologists (59 patients), neurosurgeons (54 patients), and other specialists (14 patients), as shown in Figure 1. Only 148 patients continued their follow-up in the center, and 88 patients completed a DA regimen of more than 24 months. Although all patients were maintained on DA during follow-up, the initial treatment modality was determined by the referring physician. Outcome evaluations for patients on DA were conducted at 6, 12, and 24 months, as well as at any point beyond 24 months (up to 36 months). The outcomes assessed included: (1) clinical improvement (resumption of regular cycle and/or pregnancy for married premenopausal women, and libido and erectile function for men); (2) normalization of prolactin; and (3) radiological changes of prolactinoma.

The number of patients included at each evaluation point reflected those who attended follow-up visits at that time. Patients with persistent high prolactin or increasing prolactinoma diameter despite being on maximum dose DA (3.5 mg/week) were referred for either TSS or gamma knife radiosurgery, provided they consented to the intervention. Patients who subsequently underwent TSS or gamma knife treatment were excluded from further DA outcome analyses. Similarly, patients who discontinued DA for any reason were excluded from later evaluations.

### Clinical evaluation

Patient histories were reviewed for age at diagnosis, presenting complaints, and comorbidities, including diabetes mellitus, hypertension, thyroid disease, or other endocrine disorders. Previous and current treatment modalities were also recorded, including TSS, gamma knife radiosurgery, and/or medical therapy.

Each patient underwent an anthropometric evaluation, including measurement of height (in meters) and weight (in kilograms), from which body mass index (BMI) was calculated. A collection of clinical complaints related to prolactinoma, including galactorrhea, oligomenorrhea, hirsutism, acne, vasomotor symptoms, infertility, headache, visual field defects, gynecomastia, reduced libido, and erectile dysfunction, was surveyed and evaluated. Remission was defined as a cessation of hyperprolactinemia symptoms, normalization of serum prolactin, and disappearance of adenoma.

### Biochemical analysis

After an overnight fast of at least 8 hours, venous blood samples were obtained from all patients. Samples were collected in tubes containing a clot activator and centrifuged immediately. Serum was analyzed for prolactin (PRL) and related hormones, including follicle-stimulating hormone (FSH), luteinizing hormone (LH), total testosterone, estradiol, adrenocorticotropic hormone (ACTH), dehydroepiandrosterone sulfate (DHEA-S), thyroid-stimulating hormone (TSH), free thyroxine (FT4), total thyroxine (T4), and growth hormone (GH).

PRL was measured from separated serum using electrochemiluminescence immunoassays on the Roche Cobas e411 platform (Roche Holding, Basel, Switzerland). For this study, the reference ranges for baseline PRL were defined as 85.1–489.3 mIU/L in men and 85.1–638.2 mIU/L in women.

### Radiological assessment

An MRI was conducted for each patient with confirmed hyperprolactinemia, both at diagnosis and during follow-up, to assess the pituitary adenoma response to DA, TSS, and gamma knife. The radiology results also helped establish baseline status at 3–6 months after TSS. The timing of MRI after treatment with DA varied based on the size of the adenoma, its proximity to the optic chiasm, and response to treatment as measured by PRL levels. Serial imaging was conducted for cases of treatment-resistant prolactinoma, as well as upon manifestation of any new symptoms, such as headache, visual change, or galactorrhea, which indicated the emergence of new pituitary dysfunction.

Sequences of pituitary MRI imaging were classified according to the international standard [11]. Adenomas were classified as microadenoma if less than 10 mm in size, macroadenoma if 10 mm or more, and giant prolactinoma (4 cm or above) [3].

### Statistical analysis

All patients with a confirmed diagnosis of prolactinoma were included in the study. Data were entered into Microsoft Excel and subsequently analyzed using the Statistical Package for the Social Sciences (SPSS) for Windows, version 23.0 (SPSS Inc., Chicago, IL, USA). Continuous variables were summarized as mean ± standard deviation (SD), and categorical variables as frequencies and percentages. Baseline serum prolactin (PRL) levels were further categorized using a cut-off value of 10,638.2 mIU/L (500 ng/mL) to evaluate their association with study outcomes. This cut-off was determined by receiver operating characteristic (ROC) curve analysis against two-year remission, which yielded an area under the curve (AUC) of 0.67 (*P* = 0.01). At this threshold, sensitivity was 96% and specificity was 42%. Associations between potential predictive factors and clinical improvement, PRL normalization, radiological response, and remission were analyzed using the chi-square test. Factors found to be significantly associated with remission were subsequently entered into a multinomial regression analysis. A *P* value <0.05 was considered statistically significant for all tests.

## Results

Table 1 summarizes the baseline characteristics of patients with prolactinoma. The mean age at diagnosis was 34.8 ± 12.4 years, with a female-to-male ratio of 1.5:1. The mean body mass index (BMI) was 32.6 ± 7.1 kg/m^2^, and most patients were obese, with BMI ≥ 30 kg/m^2^ observed in 123 of 196 patients (62.8%). Regarding clinical presentation, 112 of 122 patients (91.8%) presented with oligomenorrhea/amenorrhea, 68 of 80 (85%) had reduced libido and/or erectile dysfunction, and 145 (70.7%) presented with headache. In this study, 66.4% (117/176) of patients were diagnosed with macroadenomas (≥ 10 mm). As for interventions, 149/205 (72.6%) were treated with DA, while 41/205 (20%) underwent TSS, and 5/205 (2.4%) opted for gamma knife.

**Table 1:** Baseline characteristics of patients with prolactinoma (*n* = 205)

Variable	Mean ± SD or *n* (%)
Age at diagnosis (years)	34.8 ± 12.4
Age (years) range	16–86
Age ≥ 45 years	39 (19.0)
Gender (male)	80 (39.0)
Married	165 (80.5)
BMI (Kg/m^2^)	32.6 ± 7.1
BMI ≥ 30 (Kg/m^2^)	123/196 (62.8)
T2DM	19 (9.2)
Hypertension	38 (18.5)
Galactorrhea	84/205 (41.0)
Oligomenorrhea/amenorrhea	112/122 (91.8) *
Hirsutism	17/125 (13.6)
Acne	12/125 (9.6)
Vasomotor symptoms	4/125 (3.2)
Gynecomastia	10/80 (12.5)
Reduced libido	68/80 (85.0)
Erectile dysfunction	68/80 (85.0)
Infertility	104/154 (67.5) ^β^
Headache	145 (70.7)
Visual field defect	55/205 (26.8)
PRL (mIU/l) (*n* = 191)	27,906.3 ± 78529.7
PRL (mIU/l) median	6,234.0
FSH (mIU/mL) (*n* = 138)	5.1 ± 8.2
LH (mIU/mL) (*n* = 138)	4.1 ± 6.6
TT (nmol/l) (men) (*n* = 44)	4.2± 3.8
TT (nmol/l) (women) (*n* = 46)	4.0 ± 1.0
E2 (pmol/l) (women) (*n* = 64)	199.3 ± 262.5
ACTH (pmol/l) (*n* = 84)	7.1± 4.9
Cortisol (nmol/l) (*n* = 101)	308.9 ± 151.7
DHEA-s (µmol/l) (*n* = 92)	8.1 ± 6.3
TSH (µIU/mL) (*n* = 135)	2.5 ± 1.9
FT4 (pmol/l) (*n* = 88)	15.4 ± 12.8
T4 (nmol/l) (*n* = 17)	176.3 ± 272.8
GH (mIU/l) (*n* = 33)	4.5 ± 18.9
Adenoma diameter (mm) (*n* = 176)^µ^	16.5 ± 12.3
Adenoma diameter (mm) range (*n* = 176)	3–76
Adenoma < 10 mm	59/176 (33.5)
Adenoma ≥ 10 mm	117/176 (66.4)
Adenoma ≥ 40 mm	14/176 (7.9)
Cystic adenoma	29/176 (16.4)
Cavernous invasion	53/176 (30.1)
Optic chiasm abutment	41/176 (23.3)
Hydrocephalus	3/176 (1.7)
Empty Sella syndrome	14/176 (7.9)
Apoplexy	5/176 (2.8)
Initial treatment (*n* = 205) No treatment	10 (4.8)
DA	149/205 (72.6)
TSS	41/205 (20.0)
Gamma knife	5/205 (2.4)
Continuing DA at FDEMC (*n* = 148) Patients on cabergoline	115/148 (77.1)
Cabergoline (mg) per week	0.7 ± 0.4
Patients on bromocriptine^£^	34/148 (22.9)
Bromocriptine (mg) per day^£^	3.0 ± 1.5

* Exclude three postmenopausal women. β Within married and childbearing. µ Five non-tumoral cases are not included. £ Shifted to cabergoline after six months. Abbreviations: SD, standard deviation; kg, kilogram; m2, square meter; PRL, prolactin; FSH, follicular stimulating hormone; LH, luteinizing hormone; TT, total testosterone; E2, estradiol; ACTH, adrenocorticotrophic hormone; DHEA-s, dehydroepiandrosterone sulphate; TSH, thyroid-stimulating hormone; FT4, free thyroxine, T4, total thyroxine; GH, growth hormone; DA, dopamine agonist; TSS, transsphenoidal pituitary surgery.

**Figure 2:**
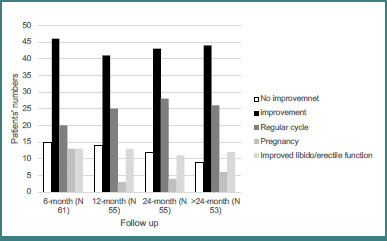
Clinical improvement in patients with prolactinoma during follow-up

Figure 2 shows clinical improvement parameters over more than 24 months of follow-up. Among female patients, 25 (50.0%) resumed regular menstrual cycles by 24 months, while 13 (21.3%) achieved pregnancy within the first 6 months of treatment.

**Figure 3:**
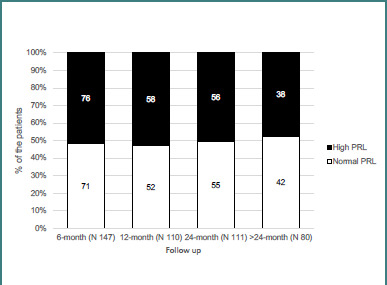
Normalization of serum PRL levels in patients with prolactinoma during follow-up

Figure 3 shows the normalization of serum PRL over time. Seventy-one of 147 patients (48.2%) achieved normal PRL levels after 6 months of treatment. At the 24-month mark, that number was 42 out of 80 (52.5%).

**Figure 4:**
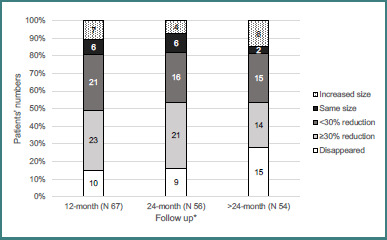
Changes in maximum prolactinoma diameter during follow-up. Over the course of follow-up, additional radiological findings included empty sella in 22 patients, cystic changes in 18 patients, chiasmal prolapse in 8 patients, and apoplexy in 2 patients.

Figure 4 illustrates changes in prolactinoma size over time. After 12 months of treatment, adenoma shrinkage greater than 30% was observed in 23 patients (34.3%), while complete disappearance of the adenoma occurred in 6 patients (8.9%).

Table 2 presents the effect of various factors on clinical improvement during follow-up. Neither patient sex nor age at diagnosis showed a significant correlation with clinical improvement across time points. Baseline serum PRL <10,638.2 mIU/L (500 ng/mL) was significantly associated with earlier clinical improvement at 6 months (*P* = 0.002) and 12 months (*P* = 0.006). Patients with adenoma size of less than 40 mm were more likely to have clinical improvements at 12 and 24 months (*P* = 0.001 and 0.003, respectively). Regarding initial treatment, patients in the DA group were more likely to experience clinical improvements at 6 and 12 months (*P* values of 0.006 and 0.001, respectively).

**Table 2:** Association of clinical improvement during follow-up with patient- and disease-related factors in prolactinoma

Factors	Clinical improvement *n* (%)			
	6 months	12 months	24 months	>24–36 months
Men	13/18 (72.2)	18/26 (69.2)	11/17 (64.7)	12/15 (80.0)
Women	33/43 (76.7)	38/50 (76.0)	32/38 (84.2)	32/38 (84.2)
*P* value	0.7	0.5	0.1	0.7
Age at diagnosis < 45 years	41/54 (75.9)	47/64 (73.4)	38/49 (77.6)	37/46 (80.4)
≥ 45 years	5/7 (71.4)	9/12 (75.0)	5/6 (83.3)	7/7 (100)
*P* value	0.7	0.9	0.7	0.09
Baseline PRL < 10,638.2 mIU/l (500ng/ml)	39/45 (86.7)	45/54 (83.3)	30/37 (81.1)	31/36 (86.1)
≥ 10,638.2 mIU/l (500 ng/ml)	7/15 (46.7)	11/21 (52.4)	13/18 (72.2)	12/16 (75/0)
*P* value	0.002	0.006	0.45	0.3
Adenoma diameter <10 mm	19/22 (86.4)	23/26 (88.5)	16/17 (94.1)	12/15 (80.0)
10–39 mm	23/31 (74.2)	29/40 (72.5)	27/34 (79.4)	23/27 (85.2)
≥ 40 mm	1/4 (25.0)	0/5 (0)	0/3 (0)	3/5 (60.0)
*P* value	0.03	< 0.001	0.003	0.3
Initial treatment DA	38/45 (84.4)	48/58 (82.8)	37/45 (82.2)	40/46 (87.0)
TSS/gamma knife	8/16 (50.0)	8/18 (44.4)	6/10 (60.0)	4/7 (57.1)
*P* value	0.006	0.001	0.1	0.05

Abbreviations: PRL, prolactin; mm, millimeter; DA, dopamine agonist; TSS, transsphenoidal pituitary surgery.

Table 3 shows the effect of factors on normalization of serum PRL over the follow-up period. There was no correlation between the patients’ gender and normalization of serum PRL. Patients diagnosed at age 45 or older were significantly more likely to have a normal PRL level at 24 months (*P* = 0.009). Patients with an adenoma size of less than 40 mm were more likely to have normal PRL after 6 months (*P* = 0.006). However, initial treatment and baseline PRL did not correlate significantly with the normalization of serum PRL.

**Table 3:** Factors associated with normalization of serum prolactin during follow-up in patients with prolactinoma

Factors	Normal PRL *n* (%)			
	6 months	12 months	24 months	>24–36 months
Men	29/60 (48.3)	32/62 (51.6)	24/49 (49.0)	21/32 (65)
Women	42/87 (48.3)	44/93 (47.3)	31/62 (50.0)	21/48 (43)
*P* value	0.9	0.6	0.9	0.06
Age at diagnosis < 45 years	57/116 (49.1)	60/124 (48.4)	38/88 (43.2)	30/63 (47.6)
≥ 45 years	14/31 (45.2)	16/31 (51.6)	17/23 (73.9)	12/17 (70.6)
*P* value	0.6	0.7	0.009	0.09
Baseline PRL < 10,638.2 mIU/l (500ng/ml)	53/100 (53.0)	59/108 (54.6)	34/72 (47.2)	29/51 (56.9)
≥ 10,638.2 mIU/l (500 ng/ml)	16/43 (37.2)	16/43 (37.2)	20/37 (54.1)	12/24 (50.0)
*P* value	0.08	0.053	0.4	0.5
Adenoma diameter <10 mm	25/43 (58.1)	28/46 (60.9)	18/29 (62.1)	13/17 (76.5)
10–39 mm	38/79 (48.1)	37/82 (45.1)	30/65 (46.2)	19/45 (42.2)
≥ 40 mm	1/13 (7.7)	3/13 (23.1)	5/10 (50.0)	5/10 (50.0)
*P* value	0.006	0.03	0.3	0.055
Initial treatment DA	57/112 (50.9	60/119 (50.4)	45/88 (51.1)	34/60 (56.7)
TSS/gamma knife	14/35 (40.0)	16/36 (44.4)	9/13 (40.9)	8/20 (40.0)
*P* value	0.2	0.5	0.3	0.1

Abbreviations: PRL, prolactin; mm, millimeter; DA, dopamine agonist; TSS, transsphenoidal pituitary surgery.

Table 4 demonstrates factors associated with prolactinoma diameter reduction during follow-up. Normalization of serum PRL at 12 months was significantly associated with adenoma shrinkage ≥30% at the same time point (*P* = 0.01). Furthermore, adenoma shrinkage at 12 months was significantly correlated with complete tumor disappearance at later follow-up (*P* = 0.009).

**Table 4:** Factors associated with prolactinoma diameter reduction during follow-up

Factors	Shrinkage ≥ 30%			Disappeared
	12 months	24 months	>24–36 months	
Men	14/28 (50.0)	12/23 (52.2)	13/18 (72.2)	12/43 (27.9)
Women	20/39 (51.3)	18/33 (54.5)	19/39 (48.7)	16/47 (25.4)
*P* value	0.9	0.8	0.09	0.7
Age at diagnosis < 45 years	24/49 (49.0)	24/42 (57.1)	23/43 (53.5)	24/83 (28.9)
≥ 45 years	10/18 (55.6)	6/14 (42.9)	9/14 (64.3)	4/23 (17.4)
*P* value	0.6	0.3	0.4	0.2
Baseline PRL < 10,638.2 mIU/l (500ng/ml)	22/39 (56.4)	16/35 (45.7)	23/40 (57.5)	21/69 (30.4)
≥ 10,638.2 mIU/l (500ng/ml)	12/28 (42.9)	14/21 (66.7)	8/16 (50.0)	6/36 (16.7)
*P* value	0.2	0.1	0.6	0.1
6-month PRL Normal	17/31 (54.8)	13/24 (54.2)	17/27 (63.0)	15/30 (50.0)
High	16/33 (48.5)	12/26 (46.2)	12/23 (52.2)	11/48 (22.9)
*P* value	0.6	0.5	0.4	0.2
12-month PRL Normal	25/39 (64.1)	14/26 (53.8)	18/27 (66.7)	16/51 (31.4)
High	9/28 (32.1)	13/27 (48.1)	12/24 (50.0)	11/46 (23.9)
*P* value	0.01	0.6	0.2	0.4
Adenoma diameter < 10 mm	6/14 (42.9)	5/12 (41.7)	7/14 (50.0)	6/22 (27.3)
10–39 mm	20/43 (46.5)	20/37 (54.1)	21/37 (56.8)	20/73 (27.4)
≥ 40 mm	8/10 (80.0)	5/7 (71.4)	4/6 (66.7)	2/11 (18.2)
*P* value	0.1	0.4	0.8	0.8
12-month ≥ 30% shrinkage	-	-	-	13/34 (38.2)
12-month < 30% shrinkage	-	-	-	3/33 (9.1)
*P* value	-	-	-	0.009
Initial treatment DA	22/52 (42.3)	24/46 (52.2)	23/43 (53.5)	18/82 (22.0)
TSS/gamma knife	12/15 (80.0)	6/10 (60.0)	9/14 (64.3)	10/24 (41.7)
*P* value	0.01	0.6	0.4	0.054

Abbreviations: PRL, prolactin; mm, millimeter; DA, dopamine agonist; TSS, transsphenoidal pituitary surgery.

Patients who underwent transsphenoidal surgery achieved adenoma shrinkage earlier than those treated with DA alone (*P* = 0.01).

Table 5 presents the association between remission of prolactinoma and clinical factors. Adenoma diameter was strongly correlated with remission, with smaller tumors achieving higher remission rates (*P* = 0.001). Patients with baseline PRL levels <10,638.2 mIU/L (500 ng/mL) were significantly more likely to achieve remission compared with those with higher baseline PRL (*P* = 0.001). Additionally, patients with normal PRL at 6 and 12 months were more likely to have remission. There was no significant association between adenoma shrinkage and remission.

**Table 5:** Factors associated with remission in patients with prolactinoma

Factors	Remission *n* (%) 25/88 (28.4%)	OR* (95% CI)	*P* value
Male	11/34 (32.4)	1.3 (0.5–3.5)	0.5
Female	14/54 (25.9)		
Age at diagnosis < 45 years	21/68 (30.9)	1.7 (0.5–5.9)	0.3
≥ 45 years	4/16 (20.0)		
Baseline PRL < 10,638.2 mIU/ l (500ng/ml)	24/61 (39.3)	16 (2.0–127.7)	0.001
≥ 10,638.2 mIU/l (500 ng/ml)	1/26 (3.8)		
6-month PRL Normal	16/42 (38.1)	3.5 (1.1–11.1)	0.02
High	5/34 (14.7)		
12-month PRL Normal	17/42 (40.5)	3.1 (1.1–8.6)	0.02
High	7/39 (17.9)		
Adenoma diameter < 10 mm	11/20 (55.0)	5.5 (1.8–16.2)	0.001
≥ 10 mm	12/66 (18.2)		
< 40 mm	23/79 (29.1)	0.7 (0.6–0.8) *	0.1
≥ 40 mm	0/7 (0)		
12 months ≥ 30% shrinkage	7/25 (28.0)	0.8 (0.2–3.0)	0.8
12 months < 30% shrinkage	7/23 (30.4)		
12 months ≥ 30% shrinkage + normal PRL Yes	6/18 (33.3)	1.3 (0.3–4.9)	0.6
No	8/30 (26.7)		
Initial treatment DA	20/68 (29.4)	1.2 (0.4–3.9)	0.7
TSS/gamma knife	5/20 (25.0)		

* For the cohort, no remission. Abbreviations: PRL, prolactin; mm, millimeter; DA, dopamine agonist; TSS, transsphenoidal pituitary surgery.

Table 6 summarizes the results of the multinomial regression analysis examining factors associated with prolactinoma remission. Variables that were significantly associated with remission in univariate analysis (Table 4) were included in the model, namely baseline serum PRL, PRL normalization at 6 months, and adenoma diameter. Baseline PRL <10,638.2 mIU/L (500 ng/mL) and adenoma diameter <10 mm were both independent predictors of remission (*P* = 0.03 for each).

**Table 6:** Multinomial regression analysis of baseline prolactin, 6-month PRL normalization, and adenoma diameter on remission

Predictors	OR (95% CI)	*P* value
Baseline PRL < 10,638.2 mIU/l (500 ng/mL)	9.5 (1.1–80.5)	0.03
Normal 6-month PRL	2.7 (0.7–9.8)	0.1
Adenoma < 10 mm	4.2 (1.1–16.1)	0.03

Abbreviations: PRL, prolactin; mm, millimeter.

Five out of 25 patients who underwent remission and DA discontinuation later experienced a relapse of prolactinoma (20%). Of these, three were men and two women; four were aged less than 45 years; three had microadenoma, one had macroadenoma; and three underwent only DA therapy, while the other two had TSS (one initially, and one after 48 months of DA therapy).

## Discussion

In Iraq, patients with prolactinomas are managed by different specialists, including endocrinologists, gynecologists, neurologists, and neurosurgeons. To our knowledge, this is the first study on prolactinoma outcomes in Iraq with a relatively large sample size. We describe the management outcomes of patients with prolactinoma treated with DA, TSS, and gamma knife.

Regarding epidemiology, we found that prolactinomas were more common in women (female-to-male ratio 1.5:1), and most prolactinomas were macroadenomas. However, this ratio is not consistent with other studies. Chanson *et al*. stated that microadenomas were approximately four to five times more frequent than macroadenomas, and a net predominance of prolactinomas was observed in women aged 25-44 years compared to men (a male-to-female ratio of 1:5 to 1:10) [12]. Another study reported that prolactinomas affected women between 20 and 50 years of age with a 10:1 female-to-male ratio [6], while in the current study, the female-to-male ratio was far lower. This discrepancy may be explained by local healthcare practices in Iraq, where most cases of hyperprolactinemia are initially identified by gynecologists in the context of infertility or amenorrhea. These cases are often managed directly by gynecologists without referral to endocrinology services [13].

In our survey, oligomenorrhea/amenorrhea was the most common complaint at presentation, followed by reduced libido and erectile dysfunction, headache, and lastly galactorrhea. A recent meta-analysis, by contrast, revealed that the most common presenting symptoms were oligo- or amenorrhea and galactorrhea [14].

In this study, we found that most of the prolactinoma patients were obese. This result aligns with that of Peric *et al*., who reported a high prevalence of obesity in prolactinoma [15]. Furthermore, we observed that most patients achieved clinical improvement after treatment in the form of either restoration of regular menstrual cycle, spontaneous pregnancy, or improved libido/erectile function. Many studies have shown that DA can successfully restore the gonadal function in patients with prolactinoma [16]. Lower baseline PRL and smaller adenoma size were associated with better clinical improvement. In a retrospective study, patients with giant prolactinoma were less likely to achieve clinical improvement, a finding consistent with our results [17].

Our outcomes regarding adenoma shrinkage and normal prolactin levels with a dopamine agonist regimen were consistent with previous findings in the literature. Previous studies of prolactinoma management varied widely according to different inclusion criteria and DA duration [2,18,19]. A recent study in Basrah found that in patients with prolactinoma, DA resulted in tumor size reduction in 88.88% of cases, and 63.63% achieved biochemical control. The combination of adenoma size reduction and biochemical control was achieved in 58.82% of patients [20]. According to Verhelst *et al*., 31% of evaluable patients experienced an adenoma reduction of ≥ 50%, while 77% achieved biochemical control after 28 months of DA treatment [21]. In a different retrospective study, DiSarrno *et al*. found a significant (> 30%) adenoma shrinkage in 89% of patients, and normal PRL in 82% [22]. In a prospective study involving 26 patients with macroadenoma, 80.7% achieved biochemical control within 6 months, and 92.1% of patients saw adenoma shrinkage after 3 years of DA treatment [23].

Furthermore, to assess factors that can predict remission, we found that a baseline PRL level of less than 10,638.2 mIU/l (500 ng/mL) and the presence of a microadenoma independently predicted remission. In contrast, age, gender, initial treatment, adenoma shrinkage, and normalization of PRL did not predict long-term remission.

Our findings are consistent with those of Kim *et al.*, who evaluated 44 of 734 patients with prolactinoma who discontinued DA therapy after at least 12 months of treatment and normalization of PRL levels. They identified higher baseline PRL levels and cavernous sinus invasion as unfavorable predictors of recurrence [24]. Similarly, Tirosh *et al*. reported in a single-center, historical prospective study of 71 men with pituitary macroadenomas and hyperprolactinemia that a decrease in serum PRL after 6 months of DA therapy was a strong predictor of long-term PRL normalization. The authors suggested that differences may influence this finding in dose-escalation protocols for DA [25].

In our study, adenoma shrinkage showed a poor correlation with remission, which contrasts with several previous reports. Lee *et al*. found that adenoma shrinkage, defined as a tumor volume reduction >25% after 3 months of DA therapy, was a reliable predictor of response in a cohort of 44 patients with prolactinoma [26]. In another retrospective longitudinal study on 185 patients with prolactinoma by Biagetti *et al*., tumor volume shrinkage of 30 % after 3–4 months of DA therapy could predict long-term response, defined as ≥50% tumor volume reduction, with an AUC of 0.95 (CI, 0.76–0.99) [27].

Lastly, in this study, we evaluated adenoma shrinkage following DA therapy. Colao *et al*. used a similar methodology, following adenoma shrinkage over more than 3 years of DA treatment. They found that DA resulted in adenoma shrinkage in 92.3% of patients and that PRL level was a good predictor of adenoma reduction [23]. However, their study differed from ours in two important aspects. First, many of their participants had already received DA therapy prior to enrollment; only 26 of the 110 patients were treatment naive. Second, within this naive subgroup, two patients had previously undergone unsuccessful TSS.

This study has several limitations. First, the exact duration of DA treatment could not be determined for some patients, as many women had already initiated therapy prescribed by gynecologists before presenting to our center. In some cases, the lack of response was attributed to medication noncompliance and infrequent follow-up visits to FDMEC rather than issues with the DA itself. Additionally, the cost of DA and the expense associated with laboratory and radiological investigations contributed to infrequent monitoring and follow-up.

## Conclusion

DA remains the cornerstone of prolactinoma treatment in our region, demonstrating high efficacy in normalizing serum PRL levels, reducing adenoma size, and improving clinical symptoms. We found that DA could be effectively used even for invasive giant prolactinoma, although the sample size was relatively small (*n* = 14). We found that lower baseline and microadenoma independently predicted remission, while age, gender, initial treatment, adenoma shrinkage, and normalization of PRL did not predict long-term remission. Our study suggests that patients with prolactinoma should be monitored regularly after discontinuing DA to check for relapse.

## Personal thanks

The authors are grateful to the medical and laboratory personnel of FDEMC for their assistance.
